# Tradeoffs in automated financial regulation of decentralized finance due to limits on mutable turing machines

**DOI:** 10.1038/s41598-024-84612-9

**Published:** 2025-01-24

**Authors:** Ben Charoenwong, Robert M. Kirby, Jonathan Reiter

**Affiliations:** 1 INSEAD, Asia Campus, Singapore, 138676 Singapore; 2 Kahlert School of Computing, University of Utah, Salt Lake City, 84112 USA; 3 ChainArgos, Research, Singapore, 179094 Singapore

**Keywords:** Computer science, Engineering

## Abstract

We examine which decentralized finance architectures enable meaningful regulation by combining financial and computational theory. We show via deduction that a decentralized and permissionless Turing-complete system cannot provably comply with regulations concerning anti-money laundering, know-your-client obligations, some securities restrictions and forms of exchange control. Any system that claims to follow regulations must choose either a form of permission or a less-than-Turing-complete update facility. Compliant decentralized systems can be constructed only by compromising on the richness of permissible changes. Regulatory authorities must accept new tradeoffs that limit their enforcement powers if they want to approve permissionless platforms formally. Our analysis demonstrates that the fundamental constraints of computation theory have direct implications for financial regulation. By mapping regulatory requirements onto computational models, we characterize which types of automated compliance are achievable and which are provably impossible. This framework allows us to move beyond traditional debates about regulatory effectiveness to establish concrete boundaries for automated enforcement.

## Introduction

Decentralized finance (DeFi) fundamentally transforms traditional financial systems by eliminating the need for trusted intermediaries. Instead of trust in financial institutions and various intermediaries, DeFi relies on technological advances to facilitate economic transactions without centralized service providers. For example, smart contracts can be programmed to assume the roles of custodians, central clearinghouses, and escrow agents. These contracts are stored as code on public blockchains and executed as part of the system’s consensus rules and computation engine. Specific protocols can be designed to prohibit intervention and manipulation and have been deployed to replicate numerous financial services such as lending markets, exchange protocols, financial derivatives, and asset management^1–3^.

As these new developments use computational systems, they must be bound by existing results from computation theory. In this paper, we extend results by focusing on specific key features of programming languages to study a desired feature in practice: What types of DeFi systems can be proven compliant with existing financial regulations? This question is important for two reasons. First, DeFi operates outside traditional financial systems and across borders, so jurisdictional and enforcement issues arise^4,5^ that are reminiscent of considerations with the development of the internet^6^. Second, the lack of a central authority and the anonymity or pseudonymity of transactions add to the regulatory complexity^7^. Even though recent research studies whether much of DeFi is genuinely decentralized^2,8,9^, regulators still face increased technological complexity in this new financial system.

Further, the advances in these financial technologies may broaden the scope of regulatory enforcement by allowing participants to observe the rules and verify that everything is executed accordingly^10^. For example, a public immutable blockchain contains all transactions among different parties, potentially allowing better tracing of funds. We explore the possibility of DeFi complying with existing financial regulations, highlighting the challenges and opportunities presented by this innovative and disruptive technology and key features of the innovation driving specific results.

Viewed through the lens of an automated system run on a Turing Machine, financial regulation means banning specific state transitions and sequences of letters on the tape. Our choice of the Turing Machine model is deliberate as it simple and serves as “a canonical model of computation used by theoreticians to understand the limits on serial computation“ and “also serves as the primary vehicle for the classification of problems by their use of space and time^11^.” Further, we can rely on powerful results that this model of computation is sufficient to compute anything computable on what is conventionally know as a computer. We can also use related results that while simple modifications like parallelization, additional compute units or extra storage facilities may speed up a Turing Machine’s ability to complete a given task they cannot empower one to complete formerly non-computable tasks given unbounded time. As we will see these extensions map nicely on to more complex economic models and by starting from the Turing Machine we can easily extend our results across different approaches.

While our analysis focuses primarily on mechanically verifiable rules like transaction restrictions, we acknowledge that financial regulation encompasses a much broader spectrum of tools and objectives. As scholars like^12^ have shown, regulatory frameworks often serve to constitute and shape financial processes, not merely restrict them. Central banks, for instance, actively use private financial institutions to implement monetary policy rather than simply constraining their behavior. Our results on automated compliance should thus be understood as addressing a specific, though important, subset of regulatory challenges.

Taking this framework, we draw upon techniques and results from computation theory concerning the connections between different models of computing and language classes^13–16^. Because of our focus on DeFi, instead of simply considering static collections of finite state machines, strings, and other basic building blocks, we will insert a “system update” facility into the machine. In other words, how does allowing updates akin to the publication of new smart contracts affect externally imposed regulations? Since Alan Turing’s seminal work in 1937, computer scientists have known that an automated process for verifying all clearly-defined properties of generic algorithms is not possible (this result is known as “the halting problem”). However, there are circumstances under which certain features, like a given static program halting in finite time, using less than a certain amount of memory, or never accessing a particular storage location, can be proven. Similarly, it is possible to construct models under which such properties can be preserved across modifications to the code. The key is to restrict those modifications suitably.

We explore how limits on this sort of update facility can provide transitivity of properties across modifications. DeFi features some computationally challenging properties: (1) Turing-complete programming, (2) permissionless access to both transact and publish code and (3) selectively immutable code. The permissionless mutability of the system combined with the Turing completeness motivates our inquiry. A system running Turing-complete code where updates can be published permissionlessly cannot make any guarantees about its future behavior, a conclusion from early work on Universal Turing Machines (UTM)^17^.

Although a general version of “compliance” can never be achieved in DeFi, we show that it is possible to construct both (1) classes of algorithms that can make credible promises and (2) restricted update mechanisms that enable credible promises. In other words, DeFi platforms can provide compliant services like traditional centralized providers through fully automatic mechanisms. But achieving such compliance comes at a quantifiable cost within the theory of computation. The goal here is to assemble well-worn tools from computer science to show how one can construct an automated economic system that can credibly comply with a given legal system.

The remainder of this paper is organized as follows: We first present our methodological framework for analyzing automated financial systems. We then systematically examine all possible combinations of system features to identify which configurations permit meaningful regulation. After the analysis, we explore the theoretical implications and practical consequences of our findings. Finally, we conclude with recommendations for regulatory system design that acknowledge these fundamental constraints.

## Defining compliance

What exactly constitutes compliance in a computational system? Consider an economy modeled as a Turing Machine, where the machine’s state corresponds to the state of the real economy. We formalize compliance as a property of system state transitions that can be verified mechanically, following Theorem 5.8.5 of Savage^11^. Specifically, a compliant system is one where no sequence of permitted operations can result in a state that violates predefined regulatory constraints set by an external regulator. For example, if a regulation prohibits transactions with certain addresses, compliance means no sequence of permitted operations can transfer value to those addresses, either directly or indirectly. Similarly a regulator may impose requirements on intermediaries transacting in certain assets or products akin to depository receipts for those assets. Compliance would then require ensuring one does not unknowingly transact in “products akin to depository receipts” for a given list of assets.

Further, these requirements can extend beyond the assets themselves. A regulator may require that the price of an asset on automated trading platforms remains within a given band. In this case the banned state transition is an exchange of that asset for some other asset outside of the band.

These examples cover basic anti-money-laundering, securities and foreign exchange control regulations. Our formalization maps naturally to both computation theory and practical regulatory requirements, providing a rigorous framework for analyzing what properties can be guaranteed in an automated financial system. We call the set of properties to be enforced at time *t* $P_t$.

This definition maps naturally to our Turing Machine model: banned states (such as having sent funds to a prohibited address) represent violations of properties in $P_t$, and a compliant system must prove that no sequence of operations can reach these states. Unlike simple blacklisting, which only checks individual transactions, our framework requires proving that no combination of permitted operations can circumvent the regulatory constraints.

This formulation maps to well-known results in computability, such as the Halting Problem and the more general impossibility known as Rice’s Theorem: No algorithm exists to determine from the description of a [Turing Machine] whether or not the language it accepts falls into any proper subset of the recursively enumerable languages^11^. In other words, we cannot categorize arbitrary programs into specific subsets automatically and reliably. In financial regulation, the canonical “proper subset” is a ban on interacting with a given address: interactions involving a banned address are forbidden, and the acceptable subset of states includes no such transfers. By “compliance” we refer to conditions imposed by an external source that sets the financial regulations.

Based on these definitions, we immediately see that in systems offering complete anonymity, like Monero^18^ or Zcash^19^, transactions can only be classified as being within or outside the system by design. No proper subsets exist before considering a computational model. Our boundaries would apply to the extent that users unintentionally leak information within these systems.

To develop intuition, consider a hypothetical rule given by a regulator that you cannot interact with a “mixing service” like Tornado Cash^20^. That prohibition describes the service as:

[A] virtual currency mixer that operates on the Ethereum blockchain and indiscriminately facilitates anonymous transactions by obfuscating their origin, destination, and counterparties, with no attempt to determine their origin.

Based on the regulation, can we achieve compliance through automation? Unless the definition of mixing service is “smart contract which matches a given chunk of code exactly,” the answer is “no” because any helpful definition will be a “proper subset” of all possible programs. Note that a code chunk is not a Turing Machine description. Even determining whether two different chunks of code describe the same Turing Machine is generally blocked by the same results. Note that this differs from simply evaluating identical code. In other words, suppose we have some program $a \in RE$ (standing for “recursively enumerable”^11^). We know immediately that any other copy of *a* is the same program and in the same subsets of *RE*. If $a = b$ and $a \in X \subset RE$, then $b = a$ and $b \in X \subset RE$. These are identities in set theory.

In contrast, evaluating whether different codes describe the same Turing Machine is slightly different. Given $a, b \in RE$, do *a* and *b* describe the same Turing Machine? Further, perhaps we are also given $a, b \in Mixers \subset RE$. Unless $Mixers = RE$, solving this is blocked by Rice’s Theorem. And if $Mixers = RE$, we cannot say they are identical—we are just noting they are both valid Turing Machine descriptions.

However, suppose a regulator bans interactions with specific enumerated “mixers.” Although evaluating if some code is a mixer does not require classifying arbitrary code blocks, there are still two issues. First, this severely limits the regulator’s power from regulating a mutable set of protocols to only specific ones. In other words, what is often called “principles-based” regulations (as opposed to rules-based regulations)^21,22^ are impossible. We cannot ban “mixers” generally – we can only ban “mixers A, B and C.” In some sense, this is akin to banning specific means of murder rather than simply banning murder, no matter the means.

Second, and more importantly, we cannot enforce even these more straightforward rules reliably. Consider these steps:

1. Deploy a new, confusingly-coded, “mixer” labeled X
2. Send funds to the mixer X
3. Withdraw from the mixer X and feed into the mixer A
4. Withdraw from the mixer A and feed into the mixer X
5. Withdraw from the mixer X and spend freely

This procedure works because we cannot identify arbitrary mixers, so we are free to deploy and then use them before they get put on the banned list. As a result, the regulator cannot even ban all interaction with enumerated mixers – it can only reliably ban some forms of interaction. This result is a severe limit on regulatory power.

If we consider that compliance exists in an automated form, operating on publicly available data in real-time, anyone accepting that final transfer must operate in a compliant fashion. If, instead, the plan is to decide these things later based on non-mechanical analysis, we are simply operating a conventional legal system with some more computers involved. Concretely, if that last transfer can be ruled illegal after the fact, it was never an automated financial system.

Finally, reconsider this example where rather than banning “mixer A,” the rule concerns some proscribed parties (terrorists, sanctioned groups, etc). If you cannot reliably ban mixers, then the process above shows you how to fund and accept funds from those groups. Similarly a process which generates assets akin to depository receipts works to evade securities-like regulations and a byzantine way of recording trades evades price regulations.

## Methodology

We address regulatory challenges with the following method:

1. Develop a formal programming language model for a financial system built on smart contracts.
2. Define a “regulator” in a way connected to the real world.
3. Enumerate all the cases admitted by our model and derive the properties of a regulator for each case.
4. Connect sets of cases to real-world regulatory designs.

Our approach lets us cleanly map well-known and long-accepted limits on computation onto financial regulation questions that have historically been viewed from a social science perspective. The point is that questions regarding the power or authority of some judicial, regulatory, or political body are fundamentally different from those surrounding specific computation models. From Gödel’s seminal work in the 1930s, we know that not every true statement in arithmetic can be proven, yet the field of mathematics continues to progress. In a similar vein, our computational impossibility results do not render any sensible definition of compliance out of reach. But they do force regulators and system designers to weigh trade-offs and make choices.

If an economy is run on a computer, certain desirable legal outcomes may not be possible as certain types of computer designs can only compute some functions. For a computer scientist, this statement is unsurprising. We acknowledge that working through the cases of our model does not add much to the theory of computation literature; however, it provides a straightforward way of mapping across fields. While legal, economic, and political science research studies the completeness of contracts and various governmental systems, they typically do not strive to achieve mathematical precision. In contrast, we can make mathematically precise statements about the power of fully automated governance structures.

At the same time, regulators and financial system actors have long relied on procedures to freeze funds while issues are resolved. This can include additional bank holidays, legal actions, or taking certain actions on weekends. For example, bank regulators may seize, close, and transfer failed banks on weekends to avoid disrupting the normal course of business. The goal is to reopen a new, solvent successor institution on Monday morning and deal with the problems without blocking insured depositor access. Such ad hoc tools may be unavailable when a system is run mechanically. And they almost certainly are unavailable for systems like Bitcoin^23^ which lack any central operator or control mechanisms. It is worthwhile to consider what sorts of automation architectures admit possible replacements.

### Stylized in-memory economy

So far, we have merely described our model economy as “automated” and have not been precise. In this section, we work through the properties of a stylized version of a common real-world smart contract platform (Ethereum) and then reduce it to a simple, classical model of computation with equivalent power. As we will see along the way, this model framing is without loss of generality for our main results. A model’s computational “power” refers to which tasks it can solve, not execution speed. For instance, finite state machines cannot parse natural languages, while Turing Machines can solve any computable problem^11^.

But a finite state machine is sufficient to build both a desktop calculator and to solve finite-sized instances of the well-known puzzle game Minesweeper. At the same time, both infinite-sized Minesweeper and Conway’s famous “Game of Life” require the more powerful Turing Machine model of computation to solve^24^.

We start by considering the economy to be a sizeable shared-memory computer where bank balances and ownership records correspond to the values stored at certain memory locations. So, some subset of the computer’s memory contains all the banking records and similar information, and then the programs that intermediate economic interactions manipulate those balances. This setup is analogous to an infinite-memory version of Ethereum^25^. Ethereum is a platform with a single, large, but finite memory space shared across all the platform’s programs. These programs, known as smart contracts, manipulate data on the platform. Smart contracts on Ethereum are capable of describing any computable function. A computable function is a technical term that means if a given problem can be solved programmatically, this language can do so. Certain problems, such as the well-known Halting Problem, cannot be solved and are known as non-computable functions. Although Ethereum includes a finite space in practice, the Ethereum Virtual Machine programming language imposes no such limits. Therefore, Ethereum’s native programming model is Turing-complete^26^.

Recall that Turing completeness is a language property; most modern programming languages allow us to write programs that require unbounded memory, even if all such programs will indeed crash when run on any real computer.

Then, we recognize that a finite-memory random access machine such as this is equivalent to a model of computation known as a Linear Bounded Automaton, and the infinite-memory version is equivalent to a Turing Machine^11^. Since we consider our economy to run on a random access machine with unbounded memory, it is equivalent to a Turing Machine. This analogy is a standard formulation in computer science where we do not impose limits on program size or complexity at the programming language level. Instead, we use the term in the same way that a natural language such as English contains infinitely long valid sentences. We will not try to write an infinite sentence, but the rules of grammar allow us to. And questions of verification therefore must handle arbitrarily long inputs and runtimes.

Therefore, an economy run on a single shared-memory computer, programmed with a standard Turing-complete programming language manipulating in-memory balances, is equivalent to an economy run on a stylized model of computation known as a Turing Machine. As we will discuss later, this model is universal in that it captures all automated economic models describable with computers as we understand them today.

## Mutable turing machine model

We need a formal model of how an automated financial system works to properly study its properties. To enable analysis, we present a simple set of algorithms run on standard computing models.

A Turing Machine’s state comprises the tape’s state and the finite state machine in the head. We can consider some encoding of the machine state and simulate it using a different Turing Machine without affecting its computational power^11^. In the same vein, consider the algorithm:
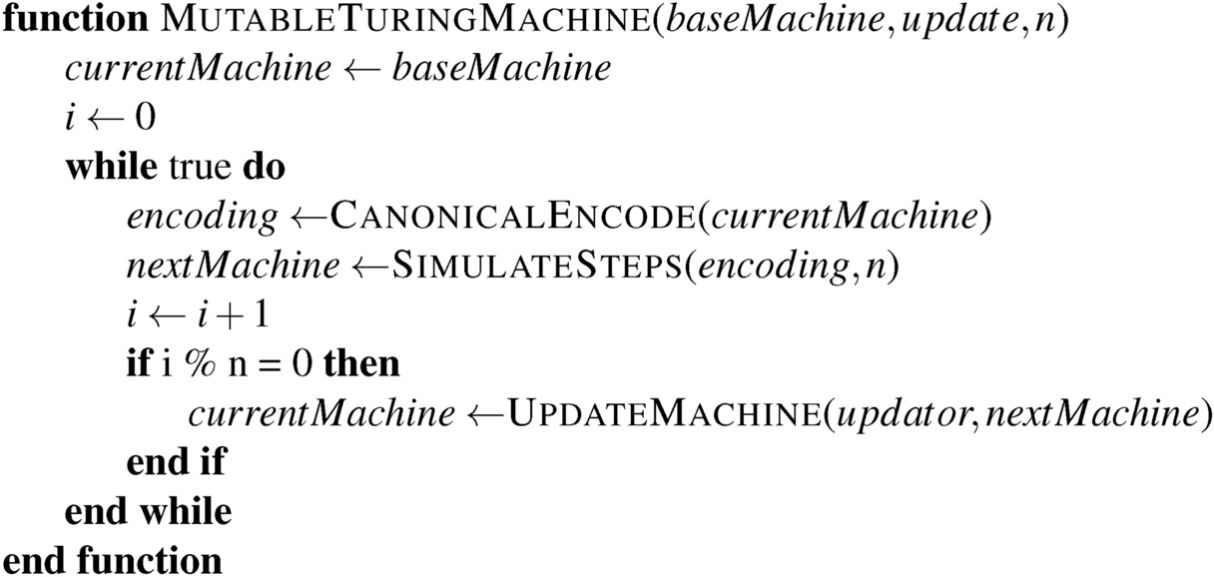
 The functions CanonicalEncode and SimulateSteps can come from^11^ or myriad other sources. We are concerned with UpdateMachine. In particular, this function works something like:
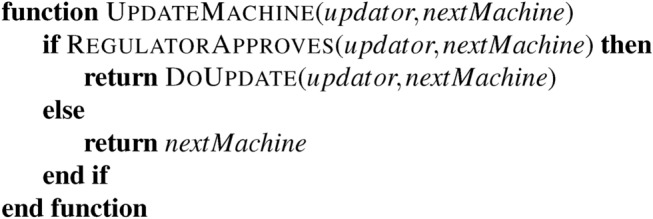
 We will now explore how this model works and show that fundamental limits restrict how powerful our updates can be if we wish to be able to write RegulatorApproves. Within this framework, a conventional permissionless blockchain^23,25^ has:
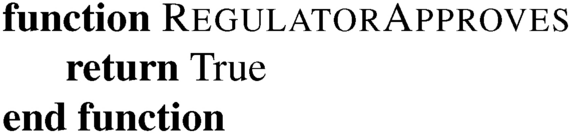
 Such products are therefore considered within this analysis. Note that this case remains consistent with Rice’s Theorem, as accepting all updates does not concern a proper subset; it concerns the entire set. We can reliably accept all updates as no classification is required.

### Initialization

This process starts with some base Turing Machine. We may well be able to prove some properties of this system using computer science tools. A proof that the following algorithm halts is straightforward:
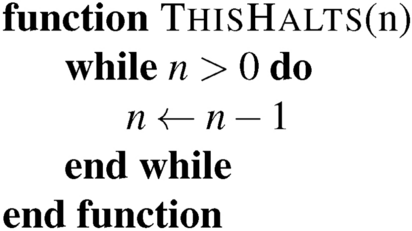
 We denote the set of proven properties of the machine at step *i* as $P_i$ with $P_0$ as the initial state before any updates run. Note that these sets can be empty.

### Updates

The next step is to consider what properties can be preserved through an update. We know that if the update process is itself Turing-complete, no mechanically-enforced rules can be followed. This means that although we can prove properties about specific update codes, we cannot make universal statements.

If the update process is described with a context-sensitive language – or something with less power – then we can prove properties about what updates are permitted. Updates are described in some language of a known-in-advance class but get generated in the future.

Notably, the UpdateMachine function can try to impose limits on these changes. For example, if updates were described with regular expressions, the update process could verify many of the properties of the proposed changes and reject those it did not like. We can think of this as some combination of a compiler and regulator. Our framework shows that the compiler has all the power for a sufficiently powerful language, and the regulator has none^27^.

### Permissions

Our motivation is to study the preservation of properties in permissionless, updateable systems. If a system is permissionless, we mean that updates can come from anywhere and anyone. What is passed into the update function gets determined anonymously in the future and is only constrained by the class of language accepted by UpdateMachine.

### Parallelization

Our model considers a single, well-ordered sequence of operations across the economy. This is a significant simplification concerning the real world, enabling us to start. For example, suppose we have two separate economies, each with financial and legal systems. In that case, the construction of an automated compliance mechanism across them is blocked by FLP impossibility^28^. This can be seen by considering how two legal or regulatory processes might interact: as long as communication is asynchronous, the FLP result applies. The assumption of synchronous communication across national regulators is unreasonable.

Further, this ability to coordinate asynchronously is reminiscent of the concept of sovereignty in international law^29^. There is no way to enforce decisions across separate sovereign jurisdictions as neither is subservient to the other’s deadlines. In a programming sense, they do not share the same clock and need not respect each other’s timeouts. International financial regulations are, therefore, not automatable so long as the system is composed of independent sovereigns. Our concern is to show that progress can be made one level down and within a single system, but at a substantial cost.

This is why we restrict our analysis to systems under a single clock and with a single well-defined ordering of operations. Whether we require a total ordering or can get by with a partial ordering depends on the precise details of the system in question as discussed in^30^. Those authors survey several transaction tracking mechanisms and find that while many require a total ordering of transactions, it is possible to construct a “bill scheme” that gets by with partial ordering. This is a decomposable ledger akin to physical bank bills.

Here, the order of operations for a single bill must be clear, but operations across bills do not always require coordination. We could consider running these on separate systems with a synchronous coordination mechanism. This flavour of extension is similar to the multi-tape or multi-head Turing Machine. And we know from^11^ that such arrangements add no power in a language hierarchy sense. Although they can make the system faster, they do not expand the space of what can be done in finite time. So, for simplicity and without losing any generality, we consider the entirety of such a scheme to run on a single Turing Machine.

### Universality of model

While our model is simple, it is universal: additional features cannot expand its computational power beyond that of a Turing Machine. This raises questions like “if the update function can edit the state in the machine’s head, can we find more power?” or “if the update function can modify the code we are simulating, can we make better promises about future behavior?” How do we know that our model is sufficiently general to handle all possible configurations?

We can consider a canonical encoding scheme for Turing Machines to be fed into a single UTM, which can simulate all others^11^. What they all have in common is they provide a way to write both state information about the world and code to interpret it next to each other on a single machine’s tape. The UTM does not further distinguish between code and state – it just runs and updates the information on the tape as required, regardless of whether it is state or code. Those distinctions may exist for us, but they do not exist for the computer processing our data.

Once we know such a machine exists, we can think of our entire algorithm as running on top of one of these simulators. A given set of regulations, balances, or any other feature of our system comprises nothing but a setup state for the simulator. It is worth being more concrete to show how the universality applies. A specific UTM construction technique is given in Section 5.5 of Savage^11^, which provides a well-specified programming language that offers access to the full power of a Turing Machine. We know that language offers the same power as any other Turing-complete programming language, so without loss of generality, let’s take that as the programming language for our economy.

Balances, trades, states, and all regulations are now written down using this language. There are no restrictions on what state or code can be updated once this is fed into the UTM. We merely know it goes ahead and runs according to those rules.

What can we say about this system? We are running on a UTM, which means, by definition, we can run any algorithm expressible in a Turing-complete programming language. We are writing code in an unrestricted general-purpose language capable of describing any Turing Machine. Consequently, we have access to the full power of this model of computation. Any additional features—self-modifying code, some complex memory arrangement, fancy programming languages with convenience features – can be simulated comfortably within our framework. The entire space of regulations expressible in full-power programming languages can be written in this simple language on the tape at the start. This does not mean our model is the most efficient way to describe or simulate an economy; however, it does mean our model captures all possible arrangements.

In some ways, this model simply shows how trying to operate an economy programmatically brings us back to familiar concepts from programming. We can think of the UTM encoding scheme as an assembly language for economic interactions. Regulators will not want to refer to a computer science theory textbook. Instead, they will express the rules in some higher-level language, which will be compiled into the machine’s assembly language.

Similarly, traders, exchanges, payment service providers, and other economic agents will likely develop their own more convenient tools for describing their businesses. This observation dates back to the earliest days of commercial computing and the origins of both COBOL and Fortran^31^ as “domain-specific” languages. These can be compiled for the same “economic machine.” This all works, and these seemingly disparate tools can talk because we know computationally they are all identical and straightforward transformations across models can be constructed. Unsurprisingly, attempts to automate an economy will likely face standard software engineering challenges. We will discuss this confrontation later when we draw a closer analogy between the practice of regulation in an automated environment and how software development teams manage code changes.

### Agent-based modelling

Our model conceives an economy as a single shared-memory computer where agents act as programs, with specific behaviors and interaction rules, ranging from simple buy/sell programs to complex market makers. This connects directly to established work in agent-based computational economics while maintaining mathematical precision. This framework is similar to those employed by researchers in “complexity” or “computational economics.”

Within this computational framework, we model agents as well-defined entities with specific behaviors and interaction rules. These agents can range from simple to complex: some might be basic programs that buy at price *X* and sell at price $Y > X$, while others could be sophisticated market makers running complex pricing algorithms. Crucially, some agents can be implemented using simple finite state machines, while others require the full power of a Turing Machine. This distinction becomes critical when analyzing what properties can be preserved across system updates.

The computational nature of our agents connects directly to established work in agent-based computational economics. We treat each agent as “a software entity within a computationally constructed world that can affect world outcomes through expressed actions^32^.” This agent-based approach fits naturally within our general framework and provides useful tools to analyze the results we will present next. By explicitly modeling these agents within our formal computational model, we can prove precise limits on what behaviors can be reliably controlled or verified.

Rather than studying equilibrium conditions, these frameworks see an economy “as not necessarily in equilibrium, its decision-makers (or agents) as not superrational, the problems they face are not necessarily well-defined, and the economy not as a perfectly humming machine but as an ever-changing ecology of beliefs, organizing principles and behaviors^33^.” As we will see shortly, the ability to constrain the update mechanism is closely tied to the question of whether or not a given system can provably comply with regulations in a general sense. But before we can use insights from agent-based models, we need to map them to our framework.

Our model conceives of the economy as a Turing Machine. Agents can exist as programs written in any class of language, including recursive enumerable languages. For example, some agents could be simulated on finite-state machines while others require the full Turing model. At the same time, we can have agents with different degrees of evolutionary power.^1^ For example, we can imagine agents that can be implemented as simple programs where only certain variable values can change via updates. A simple agent that buys goods at a constant price *X* and sells them at a constant price $Y > X$ subject to simple inventory and cash holding constraints does not require the full power of a Turing Machine. And inserting an update process that changes *X* and *Y* between rounds does not change this.

Alternatively, we might have an agent that continues to search for new trade opportunities until some complex conditions are met. And it might do so in a way that we cannot even be sure finishes in finite time. This sort of agent—which might be as simple as a potentially-non-terminating-loop around the simpler agent we just described – requires the full power of a Turing Machine.

Consider a concrete example of translating a financial regulation into our framework: the requirement that no single agent can control more than a fixed portion of a market’s volume. A simple trading agent can be implemented as a finite state machine where states represent different position sizes. With a non-Turing-complete update language, we can prove this agent’s operations will never exceed the limit. However, if we allow Turing-complete updates, an agent could deploy complex trading strategies that make such proofs impossible. This illustrates why meaningful regulation requires either restricting the update language or implementing permissions even for seemingly simple rules.

A framework for agent-based models includes seven principles. The first principle is “An agent is a software entity within a computationally constructed world that can affect world outcomes through expressed actions^32^.” Such an agent fits within our model. The other six principles explain how to construct an economy as an ensemble of such agents. These, too, fit within a general Turing Machine framework. But while our framework is relatively barren, leaving the details of the economy entirely up to the programmer, their approach of Completely Agent-Based Modelling (c-ABM) more richly maps economic concepts onto anthropomorphized computational-agent functions.

Once we establish what sorts of compromises are required in the general framework to achieve compliance, we will return to the c-ABM framework to explore what it can teach us about those compromises.

The methodology outlined above provides a systematic framework for evaluating regulatory possibilities in automated financial systems. By examining each case through the lens of computational theory, we can move from abstract possibilities to concrete conclusions about implementable regulations. The following analysis applies this framework to enumerate all possible combinations of system features and their implications for regulatory enforcement.

## Analysis

We now examine each possible combination of system features, analyzing their implications for regulatory enforcement. For each case, we consider whether meaningful regulation is possible and under what conditions. We consider eight potential systems based on the following three binary feature choices:

- Is the update language Turing-complete?
- Are updates permissionless?
- Do we start with the initial set of enforced properties $P_0 \ne \emptyset$ or $P_0 = \emptyset$

These cases are summarized in Table 1. We will analyze each case to determine whether updates in each system can always be proved to satisfy a set of externally specified rules.

**Table 1:** The various cases mapped out.

	TC, $|P_0 |> 0$	TC, $|P_0 |= 0$	NTC, $|P_0 |> 0$	NTC, $|P_0 |= 0$
Permissioned	$\hbox {Can have rules}^*$	Can grow rules*	Can have rules	Can grow rules
Permissionless	Cannot keep rules	Lawless	Can have rules	Can grow rules

*Indicates that those systems’ rules are contingent on the permissions process and no permission party going rogue rather than the system itself. TC = Turing-complete & NTC = non-turing-complete.

*Case 1. Limited Language, Permissioned,* $|P_0 |\gg 0$. Here we can design a system that ensures $P_{i+1} = P_i$. As the update mechanism is not Turing-complete, it is possible to write automated compliance checks that handle arbitrary code.

The system is also permissioned, meaning that whoever controls the permissioning can delegate compliance to the group of their choice. This system resembles a common-law legal system where designated judges interpret the rules. The system would be compliant with respect to those judges’ decisions.

Further, as the system begins with a non-empty set of rules, it can achieve compliance with respect to $P_0$ even before the first update step is run.

*Case 2. Limited Language, Permissioned,* $|P_0 |= 0$. This system begins with no rules. However, we can design an update process that allows for further initialization and imposes some rules after the first *n* steps. Such a process can also maintain compliance with these rules because the language is limited and not Turing-complete.

*Case 3. Turing-Complete Language, Permissioned,* $|P_0 |\gg 0$. We can enforce rules here, but only via the permissioning mechanism. If someone tries to publish an update that sets $|P_i |= 0$, there is no way we can mechanically stop it.

This case is akin to a real-world legal system where a group of judges can impose any changes they wish. Given they can effect arbitrary change, the only reliable mechanism to enforce long-term compliance with a given set of rules is permission.

*Case 4. Turing-Complete Language, Permissioned,* $|P_0 |= 0$. This version, again, begins with no rules. We may be able to initialize a system of regulations – but again, only through permissioning. If someone tries to publish an update that sets $|P_i |= 0$, there is no way we can mechanically stop it. We can remove their permission in the future, but as the language is Turing-complete, we cannot automate that process, and there will be some gaps during which the system is non-compliant.

Note that an underlying consensus algorithm or other process does not matter in these cases. Whoever controls the permissioning can impose their will on the system anyway. Such tools may contribute to data integrity or help with liveness or any number of other engineering challenges – but they do not extend the power of the system.

*Case 5. Limited Language, Permissionless,* $|P_0 |\gg 0$. The system is permissionless here, but the update process can impose rules. We can design a system that preserves $P_0$ through arbitrarily-many arbitrary updates by building in a process that rejects changes where $\exists p \in P_i | p \notin P_{i+1}$.

We could also design a system which ensures $|P_{i+1} |\ge |P_i |$. The rules can change here, but the number of rules never decreases over time. That is odd and likely not useful in practice. However, it shows how this formalism helps explore the space of possible regimes in a novel way.

*Case 6. Limited Language, Permissionless,* $|P_0 |= 0$. This case is closely related to the previous case. We could build an update process that ensures the rule set is brought up to some size via updates. Here, we can achieve the desired rules as $P_n$ but have no control over it before that time.

Another way to think of this case is that the first *n* steps are the initialization process. Conceptually, we could have an update process that ignores all inputs until this bootstrapping process is complete. In this sense, it is equivalent to the previous case. As we will see during later analysis, these two cases fall into the same category.

*Case 7. Turing-Complete Language, Permissionless,* $|P_0 |= 0$. This case corresponds to the initial state of a real-world blockchain. And there is no way, by Rice’s Theorem, to bootstrap a collection of system-wide rules reliably.

*Case 8. Turing-Complete Language, Permissionless,* $|P_0 |\gg 0$. This system begins with some rules. If we take $P_0$ to be our desired regulations, we are compliant for the first *n* steps. However, the system is permissionless, and updates are expressed in a Turing-complete language. So, we have no way to control the transition from $P_n$ to $P_{n+1}$ and, therefore, no way to ensure compliance beyond that time.

Again, note that a consensus algorithm cannot fix this. If we had a consensus algorithm capable of ensuring the system never exhibited a given non-trivial property, it would run afoul of Rice’s Theorem^27^ in the same manner as our mixer discussion earlier. We can formalize this using standard techniques as follows. Assume the existence of a consensus algorithm that rejects all programs *x* where $x \in Banned \subset RE$. This algorithm then classifies programs:

1. Run the algorithm among participants configured to reject members of *Banned*
2. Try to deploy *x*
3. Check if *x* was deployed
4. If so we know $x \notin Banned$ and if not $x \in Banned$

This process is precisely the classifier that Rice’s Theorem states we cannot construct. Proof-by-reduction shows that a consensus algorithm for this problem does not exist. The analysis above reveals a fundamental pattern: achieving automated compliance requires either restricting system flexibility or implementing forms of centralized control. This pattern emerges from computational theory rather than policy choices, suggesting that certain regulatory goals are fundamentally incompatible with fully permissionless, Turing-complete systems.

But that is not to say that consensus algorithms cannot solve some problems. Bitcoin’s key innovation was solving double-spending without a trusted party; however, we already had solutions for double spending that required a trusted party. And for Bitcoin, we have a constructive proof of the property in question. It is not a generic or automated scheme for enforcing arbitrary properties.

A consensus algorithm that could natively enforce compliance in our strict sense would also be able to solve the Halting Problem and therefore not be simulatable on a “merely” Turing-complete computer. If such an algorithm does exist, it requires a more powerful computation and computer design than is currently understood by science.

Having enumerated and discussed all eight cases, we can now name certain subsets and relate them to the real world. We will do this by grouping and labeling subsets of these cases in a manner reminiscent of truth tables in logic. First, all permissioned systems can achieve compliance, as shown in Table 2. Similarly, systems with non-Turing-complete update languages can also get there per Table 3. But there is no way for a permissionless system with a Turing-complete update process to achieve compliance per Table 4. This analysis of the case table tells us there are two ways to build a compliant system:

1. Permission the system, or
2. Restrict code changes to be non-Turing-complete.

We can see this in Table 5. As the underlying Turing Machine model is general and we impose no restrictions beyond these three properties, these eight cases cover all possible system designs.

Having established the feasible and infeasible cases for automated regulation, we now examine their broader implications for financial system design and regulatory policy. These results extend beyond pure theory to impose practical constraints on achievable regulatory frameworks.

**Table 2:**
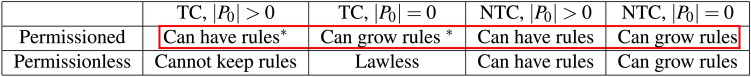
All permissioned systems can enforce rules.

*Indicates that those systems’ rules are contingent on the permissions process and no permission party going rogue rather than the system itself. TC = Turing-complete & NTC = non-turing-complete.

^**a **^All individuals coming within 1 m to inspect experimental setup and, in parentheses, all individuals that made attempts

^**b **^Proportion successful

**Table 3:**
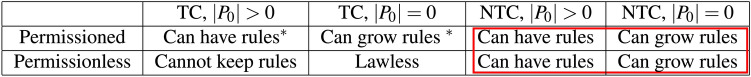
All systems with non-turing-complete updates can maintain compliance.

*Indicates that those systems’ rules are contingent on the permissions process and no permission party going rogue rather than the system itself. TC = Turing-complete & NTC = non-turing-complete.

^**a **^All individuals coming within 1 m to inspect experimental setup and, in parentheses, all individuals that made attempts

^**b **^Proportion successful

**Table 4:**
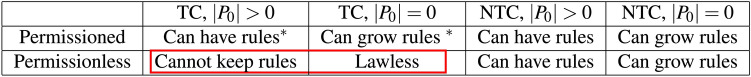
Permissionless systems with turing-complete updates cannot maintain compliance.

*Indicates that those systems’ rules are contingent on the permissions process and no permission party going rogue rather than the system itself. TC = Turing-complete & NTC = non-turing-complete.

*Indicates that those systems’ rules are contingent on the permissions process and no permission party going rogue rather than the system itself. TC = Turing-complete & NTC = non-turing-complete.

^**a **^All individuals coming within 1 m to inspect experimental setup and, in parentheses, all individuals that made attempts

^**b **^Proportion successful

## Theoretical implications

The parallel between iterative economic decision-making and computational state updates provides a natural bridge between traditional economic models and our computational framework. This connection helps explain why certain regulatory goals that seem intuitive in traditional economics become provably impossible in automated systems.

We can draw a few conclusions from this analysis by cases. The first is that permissioned systems can enforce rules across a broader design space than permissionless ones. The second and more important conclusion is that not only are permissionless Turing-complete systems unable to enforce rules over the long term reliably – but also that the compromise in update power required to bring order to a permissionless system lives within the update process verification algorithm.

Alongside this observation, we note that the initial state of a permissionless system is, in the long run, irrelevant when considering whether that system remains compliant. Even with $|P_0 |\gg 0$, we can still have a system that obeys none of those rules by step $n+1$. This finding is important as it means we must focus at least as heavily on the update mechanism as the initial conditions. In financial regulation, when we talk about “moment-to-moment” compliance, this is what it means in practice. The system must reliably maintain properties along a chain of updates.^2^

### Permissions on a permissionless system

In practice, embedding a permissioned system inside a permissionless one is possible. This embedding is an extension of the update process where some subset of states and tape symbols are associated with a protection process. While this sort of “proof by simulation” is common in computer science, it is not generally employed when analyzing economic systems. Consider the following function:
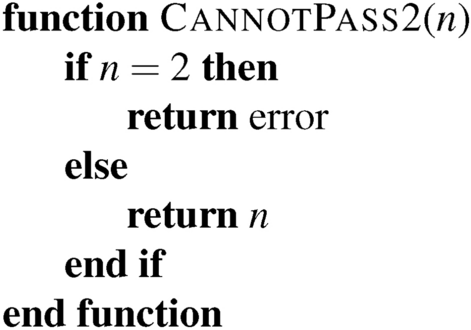
 In a Turing-complete language, we cannot determine in advance if an arbitrary block of code will call this function. No algorithm can scan arbitrary code and determine if this function is called. But we can still guarantee that, conditional on it being called, it will not return a 2. This small crack allows us to achieve compliance on restricted systems carefully.

**Fig. 1:**
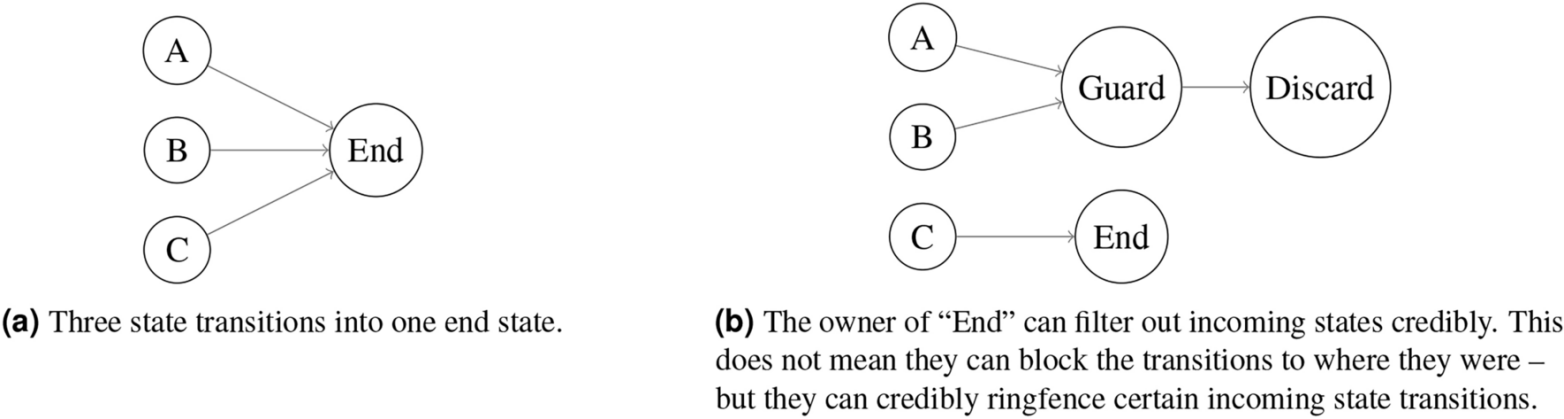
Guarding inflows transitions within a finite state machine.

Similarly, a given state within a Turing Machine’s head cannot prevent the machine from entering it via any available path in the finite state machine. But if we, as someone trying to update the Turing Machine’s logic to prevent certain state transitions, can affect this sort of local change from Fig. 1a–b we can achieve something of the sort. As we see later, these simple sorts of updates are sufficient to achieve something like a “blacklist” that prevents explicitly named state transitions.

There is a wide range of languages for describing finite state machines and tape contents. Similarly, there are many ways of describing changes to those constructs. The critical point is that if we wish to preserve properties across permissionless updates, we must choose non-Turing-complete languages.

### Real-world examples

**Table 5:**
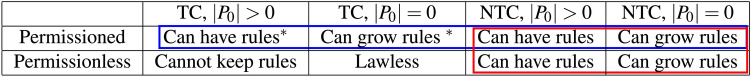
Two strategies cover all the compliant states.

*Indicates that those systems’ rules are contingent on the permissions process and no permission party going rogue rather than the system itself. TC = Turing-complete & NTC = non-turing-complete.

^**a **^All individuals coming within 1 m to inspect experimental setup and, in parentheses, all individuals that made attempts

^**b **^Proportion successful

Above, we referred to the concept of a state transition ban like “you cannot receive funds from party X.” But we also noted that it is sufficient to demonstrate that accidentally-received funds are properly ringfenced; the regulator’s demands acknowledge the realities of how the world works. Again, as discussed above, we can enforce these rules continuously with a non-Turing-complete update language.

Further, the regulatory process must know who is and is not trying to achieve compliance. If a new state transition into “End” in Fig. 1b is introduced, we must ensure it complies with the operator of the End’s rules. Compliance is a global property, and the process by which it is enforced must handle unknown code. The process may involve banning such updates or allowing the machine’s “out of bounds” portions free-reign. The former is a form of algorithmic permissioning, while the latter restricts the compliant from non-compliant portions of the state space.

To see this in practice, consider this sort of contract that we can introduce into a system
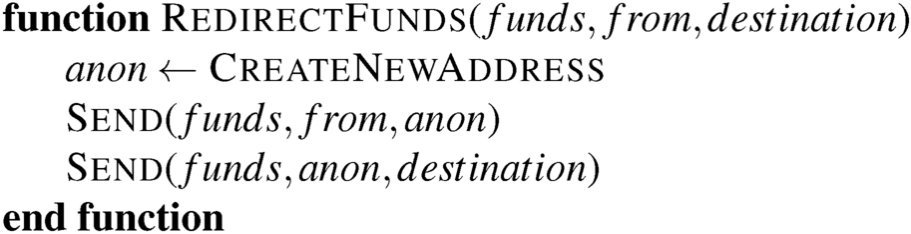
 Addresses correspond, in some sense, to locations on the Turing Machine’s tape. One key lesson of Rice’s Theorem is that we cannot guarantee what arbitrary code writes on an unbounded tape. No regulator can reliably block this sort of function without blocking all updates. The only available solution is to restrict ourselves to a weaker update language.

Let us consider introducing a new protocol with richer code into our system. If this code is Turing-complete, we cannot reject the update if it permits a prohibited state transition. In the model presented above, any working implementation of RegulatorApproves would run afoul of Rice’s Theorem.

This result follows the same argument made concerning certain proposed solutions to Ethereum’s DAO hack in 2016 in^36^. An extensive collection of tokens was “misappropriated” due to a smart contract bug. Several interested parties proposed modifying the system to prevent interaction with those tokens. Such a solution ran into the same problems, and the proposal was dropped. That long-accepted limitation is a straightforward consequence of this work.

We see here that in a restricted system, whether the restriction takes the form of permissions or reduced update power, achieving something like this level of control possible. Such control requires two things. First, the system must be compromised. Second, someone must work to prove that a given setup enforces the desired rules. This cannot be done mechanically-someone needs to prove compliance constructively.

#### Cryptocurrency market incidents

The cryptocurrency markets have seen several incidents where this lack of compliance, defined broadly, manifested in interesting ways. Each shows how a powerful update mechanism, or a potential code path to a non-terminating loop, can wreck an economic system.

##### The DAO

 An early project on the Ethereum blockchain, The DAO encountered a critical issue in 2016 when a bug led to a large transfer of tokens to an address considered malicious by many in the community^37^. This incident, whether termed a hack or not,^3^ led to proposals for recovering the lost value. Some of these proposals faced a fundamental challenge that aligns with our paper’s core argument: the impossibility of proving properties about arbitrary code without execution. This limitation, as described by^36^, effectively prevented the implementation of an algorithm to ban interactions with specific addresses in smart contracts pre-execution, illustrating our impossibility result in a real-world context.

Ultimately, the Ethereum community opted for a network “fork,” splitting the blockchain into two versions: “Ethereum,” which reversed the supposed hack through an “irregular state change”^39^, and “Ethereum Classic,” which maintained the original transaction history. This solution, which fractured the network and delegated the regulatory decision to users^39^, starkly contrasts with traditional financial regulation methods. It demonstrates the unique challenges in regulating decentralized systems and underscores the relevance of our paper’s findings on the limitations of automated compliance in Turing-complete environments.

*Beanstalk Finance.* In 2022, Beanstalk Finance experienced losses from what is now called a “governance attack”^40^. This decentralized finance protocol, like many others, includes a governance mechanism mediated by publicly visible and accessible smart contracts^41^. A stylized description of the process is:

1. There is some project token. Holding this token is required to participate in the process.
2. Anyone with a sufficient token holding can propose, publicly and with code, a change to the system.
3. Token holders can vote publicly for or against the proposal. There is some time limit.
4. The proposal passes if a sufficient number of tokens vote in favor and some lower quorum of votes is cast.
5. At this point, anyone with a token holding can call the public function which effects the changes.

A stated design goal of their mechanism was described as:

A robust decentralized governance mechanism must balance the principles of decentralization with resistance to attempted protocol changes, both malicious and ignorant, and the ability to adapt to changing information quickly. In practice, Beanstalk must balance ensuring sufficient time for all ecosystem participants to consider a Beanstalk Improvement Proposal (BIP), join the Silo, and cast their votes, with the ability to be quickly upgraded in cases of emergency.

This looks a lot like our model above. Here, the “regulator” is the set of token holders and the regulatory process is the voting.

Despite aiming to balance decentralization principles with resistance to malicious changes, the system proved vulnerable when an attacker borrowed a large share of tokens, proposed changes that would transfer the project’s treasury to themselves, and successfully passed these proposals through voting^40,42^.

This incident exemplifies the challenges our model addresses about the fundamental limitations of automated compliance in Turing-complete environments. The smart contracts governing the DAO represented a specific instance of our Turing Machine model, where: (1) the machine’s state corresponded to token balances and voting rights, (2) state transitions represented transfers and governance actions, and (3) the update mechanism allowed for arbitrary new code deployment.

It demonstrates the difficulty of enforcing externally imposed requirements (such as preventing self-serving proposals) in a permissionless system with a Turing-complete update facility. The complexity of code interacting with a dynamic economy makes it impossible to predict or prevent all potential exploits before execution reliably. This limitation applies to straightforward attacks like Beanstalk’s and more complex scenarios involving intricate trading systems or subtle manipulations. The Beanstalk example thus reinforces our paper’s argument, echoing the unresolvable nature of the DAO hack through simple programming changes.

Our impossibility results explain why no simple programming fix could guarantee the absence of exploits without fundamentally restricting the system’s update capabilities. This connection between abstract theory and practical failure helps explain why certain seemingly-intuitive fixes were mathematically impossible.

##### Compound

 In 2024, the Compound protocol faced an attack similar to Beanstalk Finance, but with a crucial difference: a multi-day delay between successful votes and upgrade deployments^43^. This delay allowed the Compound team to intervene when a malicious upgrade passed, negotiating with the “attacker” to withdraw the proposal before implementation. This incident highlights a shift from pure computer science to a political process addressing security vulnerabilities.

This case directly supports our paper’s impossibility result by demonstrating that mechanically enforcing a “no upgrades which allow theft” policy is infeasible in a Turing-complete system. The time delays and human intervention represent a compromise necessitated by the unwillingness to reduce the update facility’s richness or introduce permissions. This example underscores our argument that in permissionless, Turing-complete systems, perfect automated compliance is unattainable, and some form of external, non-automated process becomes necessary for maintaining system integrity.

##### Terra/LUNA

 The Terra project, built around the LUNA governance token and UST “algorithmic stablecoin,” aimed to maintain UST’s price at $1 through software-driven trading and stabilization strategies^44^. However, in May 2022, the system collapsed, with both tokens’ values plummeting to near zero^45^. This failure, while not immediately apparent as a regulatory issue, can be viewed through the lens of our paper’s framework: the mandate “UST should always trade at $1” is fundamentally a regulatory statement, akin to prohibitions on specific financial transactions such as “you cannot receive money from North Korea.”

This example illustrates the limitations our paper addresses. While it’s not impossible to design a $1-pegged token system, such a mechanism must be constructively proven to achieve its goal of being decidedly stable. Terra’s reliance on dynamic algorithms and upgradeable trading strategies highlights the pitfalls of depending on flexible, Turing-complete systems for regulatory compliance. As our paper argues, achieving specific regulatory outcomes often requires reducing the power of the upgrade facility.^4^

##### MakerDAO

 Another $1-pegged token system, MakerDAO employs a more complex mechanism than Terra. Users deposit collateral into “vaults” to receive dollar-pegged tokens, allowing them to leverage their assets while retaining ownership^47^. For example, a user might deposit $150 worth of some token with a floating price and receive back $100 of the dollar-pegged tokens^47^. The system aims to maintain stability by enforcing high collateralization ratios and liquidating undercollateralized positions. However, this approach faces challenges in a dynamic market environment.

Why would anyone do this? Because they are not exchanging one group of tokens for another. They can later choose to return 100 tokens and recover the initial collateral. Through this mechanism, they take on some leverage for trading purposes and still hold on to their original assets.

Through a governance mechanism similar to those discussed above, community members are able to propose the acceptance of different assets as collateral for token issuance and to adjust the “collateralization ratio” – the ratio of value deposited to the value of $1 tokens return – for each asset over time.

To keep the system stable and avoid a Terra-like collapse, the system also enforces that the value of each collateral pledge – which, recall consists of things that are not necessarily intended to have stable prices – must remain significantly higher than the number of dollar-pegged tokens originally issued against it.

But how can it do that? The MakerDAO protocol does not control all the prices in the world. Rather, it tells depositors that if their collateral value falls too close to the number of dollar-pegged tokens they withdrew, it will seize their collateral and sell it for dollars to make the system whole. If the $150 of value deposited above drops to just $$\$100 + \epsilon$$ and the original depositor does not return their 100 tokens, the system allows them to walk away and sells the collateral for dollars. A larger buffer, say $$\epsilon =5$$ rather than $$\epsilon =1$$, triggers the sale at a higher price and increases the system’s safety margin. However, as is the trend here, we cannot guarantee the outcome of trading activities in a dynamic system like this.

The property MakerDAO wants to enforce is “the collateral can always be liquidated for more money than the loan balance.” We cannot prove this will always be true as long as MakerDAO interacts with dynamic contracts in a Turing-complete environment.

In March 2022, amid COVID-19-related market volatility, MakerDAO experienced liquidation losses^48^, eventually leading to community-approved debt write-offs^49^. Some users also suffered losses when rapid price fluctuations led to collateral liquidations just before market recovery^50^. These incidents highlight the difficulty of enforcing the property that “collateral can always be liquidated for more than the loan balance” in a system interacting with dynamic contracts, highlighting our paper’s central argument about the limitations of automated compliance in Turing-complete environments.

These case studies-The DAO, Beanstalk Finance, Compound, Terra/LUNA, and MakerDAO-collectively illustrate the practical manifestations of our paper’s theoretical findings. Each example demonstrates a different facet of the challenges in implementing reliable, automated compliance mechanisms in decentralized, Turing-complete systems. From governance attacks to stablecoin collapses and liquidation issues, these incidents underscore the impossibility of guaranteeing specific regulatory outcomes without compromising system flexibility or introducing external interventions.

Our impossibility result explains why these systems ultimately resort to human intervention, time delays, or accepting certain risks. The fundamental issue lies not in the specific design of these protocols but in the inherent limitations of enforcing regulatory constraints in highly dynamic, Turing-complete environments. As our paper argues, achieving robust compliance in such systems necessitates either reducing the power of the upgrade facility or accepting that some regulatory goals cannot be mechanically enforced. These real-world examples thus provide evidence for the practical relevance and importance of our theoretical findings in decentralized finance and automated regulatory systems.

### Order of operations

Note that the permissibility of certain actions may depend strongly on their order. Consider the following sequence of events where *X* is a wallet where regulations ban interaction:

1. Publish *R* as a “repeater” contract which forwards funds to *Z*
2. Send funds to *R*
3. Update R to forward funds to *X*

Swapping the second and third operations renders the transfer impermissible. So long as our smart contract language permits the construction of non-commutative operations, this phenomenon is possible. As the simple act of transferring funds from *a* to *b* is non-commutative, this is going to be an issue in practice.

As discussed above, all operations have a well-defined total order within our framework. This means there is never ambiguity around ordering concerning the regulator, and regulations can reliably be enforced even in non-commutative operations.

The situation for end users is not as good. Say a user is connected to the system through a link with significant, and variable, latency like the internet. In the example above consider different users submitting steps 2 and 3. The regulator will see these in an order and act accordingly. However, this ordering may not be predictable for the end users. In the same way that FLP prevents us from automatically coordinating regulations across independent legal systems, it also blocks users with real-world connectivity from reliably predicting the outcome of some types of regulatory action in advance. This problem exists even before we consider how the underlying transactions are handled (i.e. if there is some complex consensus process or a central authority or anything in between).

A user can send funds that end up being blocked by the regulator when that user could not have known when the order was submitted that it would be blocked. This elevates the idea of “plausible deniability” to “provably undecideable.” The unreliability, which manifests as undecidability, stems from FLP rather than Halting. However, the question of what the user was trying to do is also undecideable, given the inability to mechanically classify updates.

## Implications for financial regulation in DeFi

Returning to Table 1, we can reframe it for whether we can have a reliable regulator.

**Table 6:** Can we achieve a reliable regulator in each case?

	TC, $|P_0 |> 0$	TC, $|P_0 |= 0$	NTC, $|P_0 |> 0$	NTC, $|P_0 |= 0$
Permissioned	Yes	Yes	Yes	After *n* steps
Permissionless	For *n* steps	No	Yes	After *n* steps

Which feasible cases in Table 6 are new and interesting? Permissioned systems are still centralized. Similarly, a system that is only sometimes compliant does not offer much comfort. But we do have one box with a “yes” that is interesting: permissionless systems that are initialized to a compliant state and have a non-Turing-complete update language.

This does not mean all such systems are compliant – but we know it is possible to build examples at the cost of restricting the richness of updates. The restriction is a non-trivial constraint, especially since weaker programming languages are rarely used in practice.

This result is quite general. Our model of an automated financial system is nothing but a composition of abstract machines. Computational power is equivalent within each class of machine. Therefore, adding a second finite-state machine or more tape cannot achieve more power. In other words, fiddling with the design will not change our results. Any automated financial system, blockchain or otherwise, fits within our framework so long as a Turing Machine can simulate the underlying model.

The implications are similarly broad. Any regulator that wishes to maintain meaningful control over an automated financial system must choose between two alternatives. First, they can run a permissioned system in much the same way finance has worked for a long time. Or they can employ a permissionless one that regulates through the automated validation of proposed updates. The latter approach is new but quite literally limits potential innovation by restricting the class of functions supported by the system. This is an unavoidable tradeoff if one wishes to enforce a regulatory regime and automate financial services.

### Regulatory workflow

Traditional financial regulation involves periodic publication of comprehensive rules and guidelines. In contrast, our model transforms this into a continuous process of reviewing code changes, similar to software development workflows. Updates are submitted, validated, and either accepted or rejected based on their compliance with system constraints.

While automation through smart contracts reduces manual checks, the regulator’s role evolves rather than disappears. Instead of reviewing individual transactions, regulators must design and maintain the automated validation rules. This introduces a new form of permissioning: rather than controlling specific transactions, regulators define the boundaries of permissible system changes. For instance, they might reject modifications that exceed certain complexity thresholds or validation timeframes.

This shift from document-based regulation to code review represents more than a technical change-it fundamentally alters how regulations evolve. Rather than comprehensive periodic updates, the system favors incremental, verifiable changes. This mirrors modern software development practices where small, frequent updates are preferred over large, infrequent ones. While permissionless updates remain possible, they require significant compromise: the update language cannot be Turing-complete, and all changes must pass automated validation through the RegulatorApproves function. This cost is non-trivial, mainly as weaker programming languages are rarely used in practice. Unfortunately, we are left with limited options once we recognize the connection between a regulator’s explicit prohibition of specific state transitions and Rice’s Theorem.

### Applying an agent-based framework

That regulatory analysis connects the functions of a financial regulator with software engineering by virtue of conceiving an economy as a computer. We can gain further insight by employing the c-ABM style to explore regulations and compromises differently.

Already, we know that the unconstrained updating of Turing-complete agents will render the system ungovernable. But it is still worth considering an economy of heterogeneous agents of known type running known programs to explore this limit. We may still be able to make some progress even if the agents are modeled in a Turing-complete language.

For example, consider an economy that consists of a large collection of simple “trader” agents that can be simulated on finite-state machines traders and a single “market-operator” that requires a Turing Machine. Say that some finite set of constants parameterizes each agent’s preferences. Furthermore, assume we can prove that the operator’s market-clearing process always terminates for all possible values of all the agent’s preferences. We can remain compliant if the update function can arbitrarily change the preferences of the traders so long as it cannot change the operator.

And by modeling the economy as a Turing Machine *where the full richness of that model of computation is employed*, we can leverage that “arbitrarily” into significant complexity. Agent models, and both economic equilibrium and disequilibrium models alike, often focus on parameterizations of the real world with tractable complexity. This is understandable when designing economic mechanisms that humans will operate. Milgrom^51^ explicitly talks about the necessity of “limiting the complexity” of auction mechanisms for human reasons. But for an economy that consists of heterogeneous computer programs interacting with each other we need not eschew a design with “so much complexity that the FCC could not successfully implement it, and bidders could not understand it^51^” because our bidders are other computer programs that will not struggle to understand their instructions.

Our general-computation model of an economy allows the c-ABM framework to stretch its legs.^32^ Those authors discuss agent rationality and stochasticity. Stochasticity concerns how wildly a trader’s parameters and algorithms change, or perhaps oscillate, from period to period. It might also mean that two traders make opposing choices when faced with the same sequence of events or that the model admits parameters where an agent does not maximize wealth, utility, or any other standard economic quantity. All of these ideas are easily expressible in programs. Often inserting a single negative sign into a utility or optimization objective function is sufficient to achieve “irrationality.” And everyone with programming experience knows how easy it is to get seemingly stochastic and irrational behavior from a computer even when considering supposedly simple programs. Constraining parameters and updates is important.

At the same time, even if we have incredibly simple traders and do not allow them to be updated at all, compliance will remain out of reach if the operator’s code can be arbitrarily changed. It is not the heterogeneity of the agent or their complexity that binds us here. It is the question of which box in the truth tables above our overall system lands in. And that classification is made easier when we bring in the ideas of c-ABM rather than working from a bare Turing Machine model and blank slate of code.

### Example real regulations

So, what can we achieve in practice? Consider a scripting language where we cannot have variables. A simple “splitting the tab” contract might look like
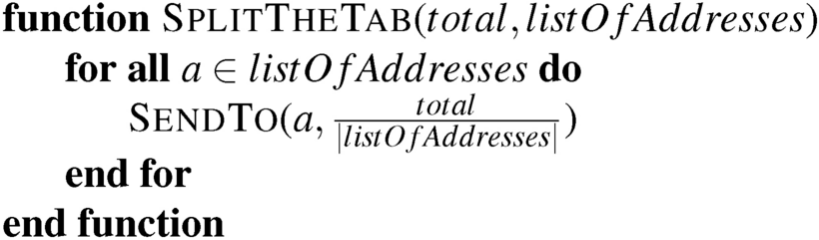
 This is dangerous if we have a regulation that certain addresses cannot be paid. The issues raised surrounding the DAO hack, discussed above, apply here. But what if the only way to transfer a token is to call SendTo and that function looks like:
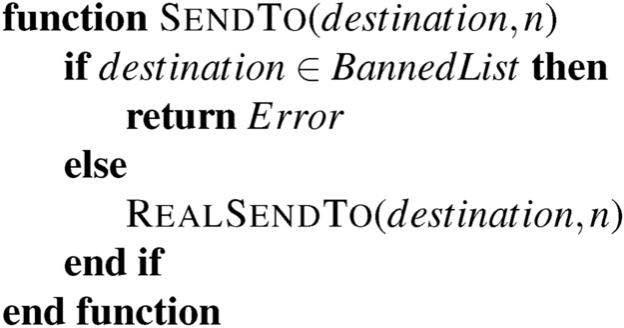
 There is no issue if a function only calls RealSendTo from SendTo. In such cases, the regulator’s responsibility is to maintain the *BannedList*, and the system is permissioned. Alternatively, we could use a permissionless system that relies on pre-2021 Microsoft Excel spreadsheets for updates. These spreadsheets are not Turing-complete^52^, and it is possible to verify in advance if a banned address is paid without the need for formal in-code gatekeeping. The regulator must then maintain the list and validate the spreadsheets manually, automatically, or through other means.

This framing of regulation represents a significant re-conception of the regulator’s role, but it is in line with what we might expect. Suppose we combine the permissionless spreadsheet-based system with the merge request analogy above. In that case, we arrive at a practical rule that sounds reasonable: do not submit overly complex spreadsheets for validation and expect approval. In essence, we have turned the financial system regulator into a code reviewer, which is again unsurprising once we make these connections.

However, things are more complex within the new paradigm of DeFi, where interactions occur via code on permissionless systems. Not all rules written down as code can be reliably implemented in DeFi. New trade-offs emerge, which regulators must confront. These are not the sorts of traditionally political or policy-related tradeoffs regulators face when weighing competing interests. Instead, some limits emerge from the computational power of the platforms themselves.

## Conclusion

This paper explores the possibility of designing a permissionless financial system that meets regulatory requirements. Although possible, creating a compliant, permissionless financial system requires compromise on the new functionality: the reliance on regulators acting as gatekeepers to ban impermissible transactions and restricting system updates to a weak programming language that can be automated to meet regulatory requirements.

Our analysis demonstrates that meaningful regulation of decentralized finance is possible, but only through fundamental compromises stemming from computational limits rather than policy choices. Creating a compliant permissionless financial system requires either restricting the system’s update capabilities to a less-than-Turing-complete programming language or implementing traditional gatekeeping mechanisms. This tradeoff is not a matter of design choice but a consequence of fundamental computational constraints.

The implications are significant for both system architects and regulators. System designers must choose between permissionless operation with limited functionality or maintaining rich features through traditional permissioned structures. Regulators, in turn, must precisely specify which rules will be mechanically enforced and commit to those boundaries, even when race conditions might temporarily allow prohibited activities. Attempting to combine unrestricted permissionless operation with comprehensive regulatory compliance ultimately reduces to traditional computer-aided financial systems, negating the core innovation of decentralized finance.

## Data Availability

The datasets used and/or analysed during the current study available from the corresponding author on reasonable request.

## Footnotes

1 Formally we can consider a set of agents and some scheduler for economic interactions which all exist as Turing Machines. As long as the agent and scheduler programs correspond to computable functions we can use standard encoding techniques to simulate the ensemble on a single UTM. Such a model imposes no limitations on the agents or scheduling process beyond that they are clearly described in a language capable of expressing any computable function.

2 This matches how compliance requirements are understood for at least some regulations in the United States. The original statement of the “Net Capital Rule” required supervised entities to comply “at all times”^34^. This was later rephrased such that the rule imposes a “moment to moment” net capital requirement^35^. Those rules apply to systems operated by humans with human supervision so there is naturally some ambiguity. In our framework we can unambiguously require that the system can never enter an impermissible state.

3 There is an ongoing debate as to whether or not making use of a feature of a decentralized finance system that the code permits but the authors publicly describe as a bug should be called a hack. That question is outside the scope of our research^37,38^.

4 Our related work^46^ demonstrates more precisely that a generic, upgradable trading strategy relying on a nebulous asset pool cannot reliably maintain a stable price, underscoring the broader implications of our impossibility result in the context of algorithmic stablecoins.
